# Differential timing of gene expression regulation between leptocephali of the two *Anguilla* eel species in the Sargasso Sea

**DOI:** 10.1002/ece3.27

**Published:** 2011-12

**Authors:** Louis Bernatchez, Jérôme St-Cyr, Eric Normandeau, Gregory E Maes, Thomas D Als, Svetlana Kalujnaia, Gordon Cramb, Martin Castonguay, Michael M Hansen

**Affiliations:** 1 Département de Biologie, IBIS (Institut de Biologie Intégrative et des Systèmes), Université LavalQuébec (Québec), G1V 0A6, Canada; 2 Katholieke Universiteit Leuven, Laboratory of Animal Diversity and SystematicsCh. Deberiotstraat 32, B-3000 Leuven, Belgium; 3 National Institute of Aquatic Resources, Technical University of Denmark2920 Charlottenlund, Denmark; 4 School of Biology, University of St. AndrewsFife, United Kingdom; 5 Fisheries and Oceans CanadaC.P. 1000, Mont-Joli, Québec (Québec), G5H 3Z4, Canada; 6 Department of Biological Sciences, Aarhus UniversityDK-8000 Aarhus C, Denmark

**Keywords:** *Anguilla*, microarray, reproductive isolation, speciation, Transcriptome

## Abstract

The unique life-history characteristics of North Atlantic catadromous eels have long intrigued evolutionary biologists, especially with respect to mechanisms that could explain their persistence as two ecologically very similar but reproductively and geographically distinct species. Differential developmental schedules during young larval stages have commonly been hypothesized to represent such a key mechanism. We performed a comparative analysis of gene expression by means of microarray experiments with American and European eel leptocephali collected in the Sargasso Sea in order to test the alternative hypotheses of (1) differential timing of gene expression regulation during early development versus (2) species-specific differences in expression of particular genes. Our results provide much stronger support for the former hypothesis since no gene showed consistent significant differences in expression levels between the two species. In contrast, 146 genes showed differential timings of expression between species, although the observed expression level differences between the species were generally small. Consequently, species-specific gene expression regulation seems to play a minor role in species differentiation. Overall, these results show that the basis of the early developmental divergence between the American and European eel is probably influenced by differences in the timing of gene expression regulation for genes involved in a large array of biological functions.

## Introduction

The North Atlantic Ocean is home to two phenotypically and ecologically similar eel species of the genus *Anguilla*: the American (*Anguilla rostrata*) and the European eel (*A. anguilla*). They both occupy various types of habitats (e.g., coastal, estuarine, and freshwater), spanning a broad latitudinal range of distribution that extends from subtropical to northern latitudes. They spend most of their lives in continental and/or coastal waters and migrate to the southwest Sargasso Sea. There, spawning of both species occurs in association with current fronts generated by the North Atlantic Subtropical Convergence Zone (McCleave 1993). Their reproduction thus largely, although not completely, overlaps both in time and space (Kleckner and McCleave 1988; Munk et al. 2010). Indeed, these two species are genetically very similar (Wirth and Bernatchez 2003) and can reproduce together; hybrids have been almost exclusively found in Iceland (Albert et al. 2006).

These unique life-history characteristics have intrigued evolutionary biologists for a long time and raised many questions that remain unanswered even today. One such question pertains to the mechanisms explaining their persistence as two ecologically very similar but reproductively and geographically distinct species, even though they are found in sympatry during spawning, which leads to potential hybridization (McCleave et al. 1987). It has long been proposed that differential development schedules during young larval stages, which subsequently influence the later timing of metamorphosis, could be a key factor determining whether young eels recruit to Europe or North America, as well as maintaining the near-perfect geographic separation between the two species (Schmidt 1925; Castonguay et al. 1994). Yet, the presence of a genetic basis underlying early developmental differences between both species has never been investigated and thus remains hypothetical.

There is increasing acceptance that variation in gene expression in levels represents a major source of evolutionary novelty, which in turn can lead to phenotypic divergence through natural selection (Wray 2007). Indeed, heritable regulatory changes in gene expression can evolve very rapidly in fish (Roberge et al. 2006) and could therefore play an important role in the early steps of species divergence (Wolf et al. 2010). In this study, we performed a comparative analysis of gene expression by means of microarray experiments involving leptocephali of American and European eels collected at the same time and in the same environment in the Sargasso Sea with the goal of documenting for the first time the transcriptional differences between the two species during early development. More specifically, we tested the alternative hypotheses of (1) differential timing of gene regulation during early development versus (2) species-specific differences in expression of particular genes involved in the differentiation of *A. rostrata* and *A. anguilla* during their early larval development.

## Materials and Methods

### Sampling and RNA extraction

Leptocephali were collected in the Sargasso Sea during the Danish Galathea III expedition in March and April 2007. A total of 33 stations distributed along three transects at longitudes 64°N, 67°N, and 70°N were sampled (Munk et al. 2010). *Anguilla*-like larvae were photographed, their length was measured, and they were immediately preserved in RNAlater™ (QIAGEN, Hilden, Germany). Species assignment to either *A. anguilla* or *A. rostrata* was carried out using three different molecular approaches based on mtDNA, nDNA, and highly variable microsatellite markers (details in Munk et al. 2010: Table S1A, see also Als et al. 2011).

Total RNA was extracted from leptocephali using the PureLink™ Micro-to-Midi Total RNA purification system (Invitrogen). RNA quality and concentration was verified using an Experion™ Automated Electrophoresis Station according to the RNA StdSens Analysis Kit protocol (Bio-Rad). A total of 48 individual larvae (24 for each of *A. Anguilla* and *A. rostrata*) were classified into four size groups (*n* = 6 for each size group; see Table 1 legend for details). Age at any given size was estimated from capture dates and length measurements, by assuming a hatch size of 3 mm, a growth rate of 0.6 mm/day until the larvae reached a length of 8 mm, followed by a growth rate of 0.38 mm/day [Jonna Tomkiewicz DTU-Aqua, personal communication; see also 13]. Admittedly, growth rate differences among these species is still debated [7, 14, 15] and has mainly been estimated in elvers and later developmental stages [16]. Consequently, the use of previously published growth rate was only meant to provide a rough approximation of age classes considered in this study and not to provide exact age determination.

**Table 1:** (A) Number and percentage of genes with statistically significant levels of expression and number of genes showing significant differences in expression levels between *A. rostrata* and *A. anguilla* leptocephali samples among four size groups (Group 1 = 10–11 mm, [mean = 10.54, SD = 0.40], estimated age = 13–16 days; Group 2 = 12–13 mm [mean = 12.58, SD = 0.51], estimated age = 18–22 days; Group 3 = 13.5–16 mm [mean = 14.46, SD = 1.20], estimated age = 23–29 days; Group 4 = 16.5–18 mm [mean = 16.71, SD = 0.84], estimated age = 29–35 days). (B) Number of genes with significant differences in expression between species that are exclusive to each size group (main diagonal); number of genes with significant difference in expression between species that are also found in other size groups (above main diagonal); number of expressed genes that are common between size groups (below main diagonal)

	Size groups			
	
	1	2	3	4
(A)				
Number of significantly expressed genes	96	106	258	222
Number of differentially expressed genes between species	9 (9.4%)	15 (14.2%)	20 (7.8%)	29 (13.1%)
(B)				
1	4 (44.4%)	2	2	3
2	78	9 (56.3%)	2	4
3	95	106	10 (50%)	9
4	89	106	189	16 (55.2%)

### cDNA microarray experiments and analysis

Since individual leptocephali samples yielded small quantities of RNA, total RNA amplification was performed prior to the standard reverse transcription protocol. About 250–300 ng of total RNA was amplified using the Superscript™ Indirect RNA Amplification kit (Invitrogen) according to the user's manual. Amplified RNA was quantified in 96-well optical plates (Corning, Corning NY, USA) using a Multiskan Spectrum spectrophotometer (Thermo Labsystems, Waltham MA, USA). A total of 500 ng of amplified RNA was retro-transcribed into cDNA using the Superscript-II protocol (Invitrogen).

The experimental design consisted in paired comparisons, where two samples of a same size group—one *A. anguilla* and one *A. rostrata*—were differentially labeled by fluorescent dyes (Cy3 and Cy5) and hybridized simultaneously on each array, for a total of 24 comparisons. Dye swaps were performed to minimize intensity-linked fluorescence biases between the dyes (Churchill 2002). Gene expression levels were measured using the ScanArray Express scanner (PerkinElmer, Downers Grove IL, USA) and quantified with the QuantArray software (PerkinElmer) using the histogram quantification method. Data spots presenting hybridization artifacts were discarded and imputed using the K nearest neighbor algorithm with 10 neighbors using the SAM software package (http://www-stat.stanford.edu/~tibs/SAM/). Raw expression data and information about the experimental design are available for download at: http://www.bio.ulaval.ca/louisbernatchez/files/eel_expression_data.zip)

The eel cDNA array used in this study was specifically developed for *Anguilla* species and contains 678 annotated genes (with unique Entrez ID), each printed in triplicates on a glass silicate matrix (details in (Kalujnaia et al. 2007)). In order for a transcript to be considered for analysis, the three replicates of this transcript had to have a significant level of expression (fluorescence intensity higher than the mean of empty spots plus twice their standard deviation (Roberge et al. 2006)) in at least one of the two samples hybridized on an array. The transcript then had to satisfy this criterion for at least one of three of the array experiments. Using this threshold, a total of 304 expressed annotated genes (44.8%) were included in the analysis. Transcription data were normalized against mean fluorescence for each dye and transformed with a base 2 logarithm prior to being corrected for intensity-related bias using the regional *R-lowess* algorithm and analyzed by analysis of variance (ANOVA) using the R/MAANOVA software package (Kerr et al. 2000) available at: http://research.jax.org/faculty/churchill/software/Rmaanova/index.html.

A mixed ANOVA model was fitted, with two random terms taking into account the variation introduced by the array (*array*) and the manufacturing lot (*batch*) as well as fixed terms accounting for the variation introduced by the dyes (*dye*), individual length (*size*), and species (*species*). Four different tests were made in order to detect the influence that species, size, and the interactions between these two have on gene expression. Namely, we assessed the presence of species effects by first considering separated size groups (six arrays per size group) and then with pooled size groups within each species (24 arrays). We also wanted to assess the presence of interaction effects by adding an interaction term (size vs. species) to the ANOVA model to identify genes showing differences in expression regulation timing between the species. Finally, we looked for size effects by pooling the two species within each size group (six arrays per size group). For all these tests, a permutation-based *F*-test *(F*s, 1000 sample permutations) was used to detect significant differences (*P* < 0.05) in transcript abundances (Cui and Churchill 2003).

Fold changes in expression levels were calculated with the R software, and in all analyses, false positive rates were estimated to correct for multiple testing using the *q*-value method (*q*-value < 0.05) (Storey et al. 2004). Exact binomial tests were conducted using the R/commander software package to test for differences in proportions of (1) genes with significant differences in level of expression between the two species and among size groups; (2) overexpressed genes in different biological processes (as explained in next section) among size groups; (3) genes from specific functional categories in both the size and interaction analyses. The proportion of overexpressed genes in one species versus the other was established for each of the functional categories by dividing the number of significantly overexpressed genes observed in each species by the number of significantly different genes between both species for a given functional category.

### Gene ontology and functional analyses

Entrez Gene IDs were obtained by converting the accession numbers of the transcripts printed on the array (based on BlastX and BlastN search results on human genome and nonredundant peptide annotation information available from NCBI (Kalujnaia et al. 2007)) using the online David Gene ID Conversion tool available at: http://david.abcc.ncifcrf.gov/conversion.jsp. Then, the online Panther Classification System gene list comparison tool (http://www.pantherdb.org/tools/compareToRefListForm.jsp) was used to identify overrepresented biological processes in the tested datasets. Namely, Fisher's exact proportion test (*P* < 0.05) was used to test for overrepresentation of biological processes among the significant transcripts compared to the representation of the same biological processes in all the analyzed genes. We also tested for functional overrepresentation by grouping related biological processes identified by Panther into seven broader functional categories that were defined using information from the SwissProt/trEMBL database (http://c.expasy.org/sprot), the NCBI browser (http://www.ncbi.nlm.nih.gov/), and completed with references from the literature (see Results section).

## Results

### Species effects

The number of genes expressed above the minimal threshold level in leptocephali of both species was 96, 106, 258, and 222 genes for size groups 1, 2, 3, and 4, respectively (Table 1A). The mean percentage of expressed genes common to any two size groups was 51% and varied between 37% and 73%, with a total of 304 analyzed genes. When the size groups were pooled, none of these 304 genes showed a significant net difference in expression between species (permutation-based *F*-test with correction for multiple tests by the *q*-value method *F*s). In contrast, differences were observed when analyzing size groups separately with the percentage of genes showing significant differences between species ranging from 7.8% to 14.2% (χ^2^; *P* < 0.05) (Table 1A). However, the number of differentially expressed genes that overlapped between any two size groups was negligible (<10%; Table 1B).

**Figure 1:**
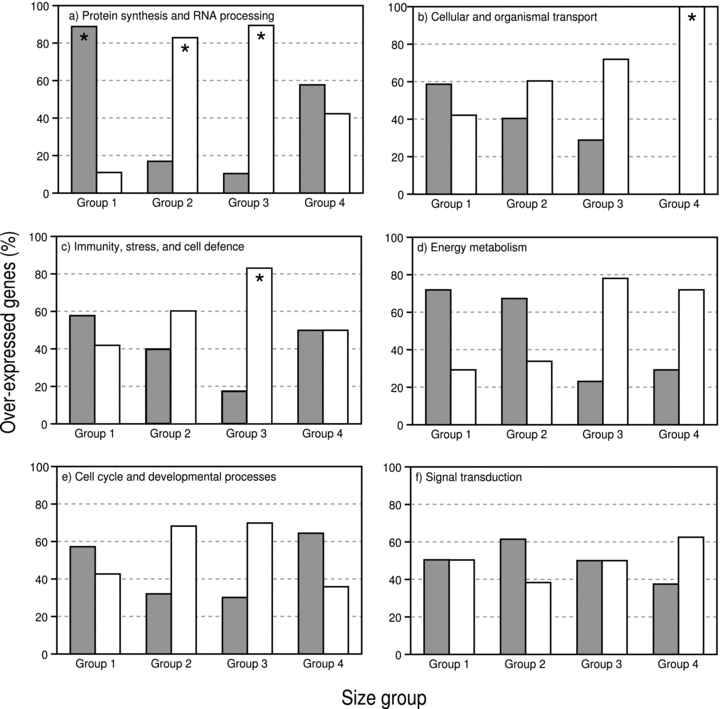
Proportions of significantly overexpressed genes in different functional categories showing variation in the timing of expression regulation between eel species. Gray bars represent proportions of overexpressed genes in *A. rostrata* and white bars in *A. anguilla*. Histograms where significantly more than 50% of the overexpressed genes are found in one species at a given size are marked with an “*” (χ2; *P* < 0.05).

Fold changes, calculated as *A. rostrata/A. anguilla* expression ratios, ranged from 0.54 to 1.36 in size group 1, from 0.63 to 2.23 in size group 2, from 0.67 to 2.09 in size group 3, and from 0.47 to 1.52 in size group 4, equating to differences in expression ranging from 5% up to 123% (details on *q*-value and fold changes in Table S1A). We then assessed whether the proportion of overexpressed genes was significantly higher than the proportion of underexpressed genes within either species and in each of the four size groups. There was a significantly higher proportion of overexpressed genes in *A. rostrata* in size group 1 (61.4%; χ^2^, *P* = 0.021) whereas the other three size groups showed a higher proportion of overexpressed genes in *A. anguilla*, although this trend was significant only for size group 3 (74%; χ^2^, *P* < 0.0001). Statistically significant differences in proportions of overexpressed genes between species (χ^2^; *P* < 0.05) were observed for protein synthesis and RNA processing functional categories in three of the four size groups, while other significant differences were also observed for cellular and organismal transport, as well as immunity, stress, and cell defense functional categories (Fig. 1).

### Interaction effects

In addition to the variation of the gene-expression fold-changes among size groups, differential timing of gene expression between species was further assessed by adding and testing for a species versus size group interaction term within the ANOVA. Here, 146 of the 304 analyzed genes (48%) showed a significant interaction effect between the species and size group terms (*F*s; *q*-value < 0.05), meaning that the timing of expression of these genes is different among the species. These 146 genes were classified into 40 distinct biological processes using the Panther Classification System. In order to eliminate the occurrence of genes in multiple categories as well as redundancy in functions generated by Panther (see Table S1 for details on gene annotations), these biological processes were grouped into seven broader functional categories: protein synthesis and RNA processing (28 genes), cellular and organismal transport (15 genes), immunity, stress, and cell defense (24 genes), energy metabolism (17 genes), cell cycle and developmental processes (26 genes), signal transduction (16 genes), and other/unclassified process (20 genes). We then tested for the hypothesis of a differential timing of regulation for these broader functional categories. A trend was observed where higher transcriptional activity was found in *A. rostrata* at earlier stages but transcriptional activity was more important in *A. anguilla* at subsequent stages. This was noted in all functional categories, except for signal transduction (Fig. 1). Namely, there was a higher proportion of overexpressed genes in *A. rostrata* in size group 1 for five of six functional categories, whereas overexpression in *A. anguilla* prevailed in size group 2 for four of six functional categories, and in size group 3 for five of six functional categories. For example, in the protein synthesis and RNA processing category, 90% of genes in size group 1 were overexpressed in *A. rostrata* whereas 82% and 92% of genes analyzed in size groups 2 and 3 were overexpressed in *A. anguilla*. For energy metabolism, 70% and 65% of genes analyzed in size groups 1 and 2 were overexpressed in *A. rostrata*, whereas 78% and 70% of genes analyzed in size groups 3 and 4 were overexpressed in *A. anguilla*. However, this pattern was more ambiguous when considering size group 4. A possible explanation for this is that the relationship between age and size is less clear as the leptocephali grow, such that individual ages among size group 4 may show more variance for their ages than the other size groups.

In the protein synthesis and RNA processing functional category, RPL5 (ribosomal protein L5; Entrez Gene ID 6125) was the most differently expressed gene (*q*-value = 0.0066, Table S1). This gene is required for 5S rRNA maturation and formation of the 60S ribosomal subunit (Frigerioa et al. 1995). It is generally involved in translational elongation but also interacts with other cellular proteins from various intracellular transport pathways (Rosorius et al. 2000). The most differentiated gene in the cellular and organismal transport category was HBZ (hemoglobin zeta; Entrez Gene ID 3050) (*qi*-value = 0.0131), which is part of the α-globin gene cluster (Brownlie et al. 2003) and plays a role in oxygen transport (Proudfoot et al. 1982) in early embryonic developmental stages (Higgs et al. 1989). Thus, higher expression of this gene in *A. rostrata* earlier in development could possibly be associated with increased aerobic metabolism needed for growth or swimming activity. Finally, the MAL gene (T-cell differentiation protein; Entrez Gene ID 4118) showed the most pronounced pattern of differentiation in the immunity and stress functional group (*q*-value = 0.0005). This gene is involved in the identification of cytotoxic/suppressor T-cells that interact with MHC class I bearing targets (Littman et al. 1985) and therefore plays a role in the immune response.

### Size effects

Of the 304 analyzed genes, a total of 60 (19.7%) showed significant differences in their levels of expression among the four size groups when data from both species were merged (*F*s; *P* < 0.05; *q*-value < 0.015). Fold changes (calculated as ratios between highest and lowest expression value among size groups for a given gene) ranged from 1.15 to 7.29, representing changes in expression between 15% and 629%. Testing for overrepresentation of biological processes using Panther (Fisher's exact proportion test, *P* < 0.05) revealed that two biological processes were significantly overrepresented (*P* < 0.05) compared to random expectation (ectoderm development, *P* = 0.023; cell structure, *P* = 0.033). It is noteworthy that none of these 60 genes were found in the previous ANOVA that included an interaction term between species and size group, suggesting that the expression level of these genes varied among size groups but that the pattern of size group related expression was similar for the two species. Thus, these genes are not involved in the differential timing of regulation observed between species.

Moreover, the proportions of genes belonging to the seven broad functional categories varied substantially between the size group and interaction analyses (Table 2). Thus, five of the seven categories showed significant differences in proportions among the results of these two analyses (χ^2^; *P* < 0.05): the “cell cycle and developmental process” (20/60; 33.3%) as well as the “unclassified biological process” (15/60; 25%) categories were overrepresented in the size analysis. In contrast, the “protein synthesis and RNA processing” (28/146; 19.2%), “energy metabolism” (17/146; 11.6%), and “signal transduction” (16/146; 11%) were overrepresented in the interaction analysis. Overrepresentation of these latter categories adds further support to their implication in the differentiation of developmental schedules among these eel species during their early larval stages. Here, the most differentiated gene for the energy metabolism group was ENO 1 (enolase 1; Entrez Gene ID 2023) (*q*-value = 0.0006, Table S1). This gene is mainly involved in glycolysis but also plays a role in growth control (Subramanian and Miller 2000), hypoxia tolerance (Semenza et al. 1996), plasminogen activity, and recruitment in inflammatory responses (Plow and Das 2009). The most differentiated gene in the signal transduction category was the pituitary form of the ADCYAP1 (adenylate cyclase activating polypeptide 1; Entrez Gene ID 116) (*q*-value = 0.0035), which is part of the cAMP-dependent signaling pathway and also acts as a hypothalamic hormone, neurotransmitter, and vasodilator (Arimura 1998).

**Table 2:** Percentages of genes belonging to the seven broad functional categories represented in the results for the size and interaction analysis (see Materials and Methods for details). Percentages correspond to the number of genes classified in a given category (numbers in parenthesis) divided by the total number of significant genes in each analysis (60 and 146 for the size and interaction analysis, respectively). The *P*-values were estimated with exact binomial tests

	Percentage (number)			
Functional category	Size analysis (total: 60)		Interaction analysis (total: 146)	*P*-value
Cell cycle and developmental processes	33.3 (20)	>	17.8 (26)	2.25 × 10^−5^
Unclassified	25 (15)	>	13.7 (20)	0.0006
Protein synthesis and RNA processing	6.7 (4)	<	19.2 (28)	4.35 × 10^−7^
Energy metabolism	5 (3)	<	11.6 (17)	0.001
Signal transduction	3.3 (2)	<	11 (16)	2.94 × 10^−5^
Cellular and organismal transport	10 (6)	=	10.3 (15)	-
Immunity, stress, and cell defense	16.7 (10)	=	16.4 (24)	-

## Discussion

The main objective of this study was to test the alternative hypotheses of (1) differential timing of gene expression regulation during early development versus (2) species-specific differences in expression regulation for particular genes in the differentiation of *A. rostrata* and *A. anguilla* during their early larval development. Our results provide much stronger support for differential timing of gene expression regulation since no gene showed a net significant difference in expression between species. In contrast, 146 genes displayed differential expression timings between the species. A generally higher level of transcription was observed in American eel leptocephali at smaller sizes whereas the pattern was reversed in larger leptocephali. The differential pattern in the timing of expression was particularly pronounced for genes involved in protein synthesis and RNA processing, energy metabolism, immunity and cell defense, as well as cellular transport. This sharply contrasted with functional categories that were overrepresented in differences among size groups (regardless of species), which mainly comprised genes of the “cell cycle and development” and “unclassified biological” processes. This indicates that the ontogeny of basic cell cycle and developmental processes is conserved in both species, whereas genes belonging to regulatory networks involved in key physiological activities (energy metabolism) and regulatory pathways (protein synthesis and RNA processing; signal transduction) have experienced ontogenetic divergence in their regulation between both species.

Overall, these results strongly suggest that differences in the timing of expression, more than species-specific gene expression differences, are at the basis of the early developmental divergence between American and European eels. Moreover, given that samples of both species were collected at the same time and in the same environmental conditions, our results provide strong evidence for genetically based temporal differences of many physiological functions between these species, in sharp contrast with their documented genetic, ecological, and morphological similarities (Avise 2003; Tesch 2003). Indeed, we found no interaction between temperature at a depth of 100 m (the most probable sampling depth of leptocephali (Munk et al. 2010)) and either species, transect, or size class, thus ruling out environmental temperature variation as a potential explanation for the observed metabolic rate differences (data not shown). This is not surprising, as no difference in vertical distribution has yet been documented between American and European eel leptocephali over their entire size spectrum (Schotch and Tesch 1984; Castonguay and McCleave 1987).

Our gene expression results seem to corroborate previous observations of faster early development of American eel leptocephali (Tesch 1998; Arai et al. 2000), and are also congruent with the hypothesis first proposed by Schmidt (1925), and later endorsed by many researchers (Castonguay et al. 1994,Arai et al. 2000; McCleave 2008; Bonhommeau et al. 2010), stating that different developmental schedules during young larval stages, which subsequently influence metamorphosis timing, could be the key factor determining the recruitment location of young eels, and thus contribute to maintaining the geographic segregation of the species. Moreover, if timing of metamorphosis in hybrids is intermediate compared to the parental species, this mechanism could explain why hybrids are almost exclusively found in Iceland, approximately halfway between the North American and European continents along the supposed larval migration route.

Establishing causal links between earlier activation of key biological functions, higher metamorphosis rates, and initiation of active migration toward the continental shelf in American eel was beyond the scope of this study. This would require circumventing the major logistical constraints linked to sampling and maintaining leptocephali alive for the duration of the experiments. Moreover, should leptocephali become available through future expeditions in the Sargasso Sea, future studies of gene expression should be based on a wider size spectrum, encompassing the full size (and age) range of leptocephali in both species, ideally between 10-mm and 60-mm long. This would allow a more comprehensive investigation of the role of differential developmental schedules on continental separation. However, American eel leptocephali reach their maximum premetamorphic size of 60 mm in about a year, whereas European eels require an estimated 2 years to reach their maximum size of 70 mm (Bonhommeau et al., 2010). This longer larval period could translate into a reduction in the average level of gene expression in larger European leptocephali than those available in this study, as metabolically more active and fast-developing American eel leptocephali leave the Sargasso Sea and metamorphose into glass eels earlier than the slower developing European eels.

Finally, differential timing of the expression level of genes implicated in such a wide array of physiological functions could play an important role in maintaining reproductive isolation between American and European eels. Namely, postzygotic isolation mechanisms, such as hybrid sterility and/or nonviability, arise as a consequence of incompatible allele combinations in hybrids that have diverged between pure species (Dobzhansky–Muller incompatibilities) (Coyne and Orr 2004). These alleles, whether neutral or positively selected for within their own genetic background, may lead to a reduction of fitness when recombined in hybrids (Burton et al. 2006). These hybrids can be unfit either due to intrinsic factors resulting in increased embryonic mortality or through external, environmentally driven factors, such as failing to find a suitable ecological niche (Burton et al. 2006). In a comparative analysis of gene expression between sympatric forms of lake whitefish (*Coregonus* sp., Salmonidae), Renaut and Nolte (2009) found that very few transcripts differed in mean expression level between embryos of the parental forms. Yet, gene expression was severely misregulated in hybrids, especially in hybrids that showed developmental abnormalities that eventually led to death (Renaut and Bernatchez). These results thus clearly revealed a persuasive link between misexpression of essential developmental genes during embryonic and larval stages and post zygotic isolation in fish. The fact that the origin of these whitefish forms is much more recent (about 15,000 years; (Bernatchez et al. 2010)) than that of American and European eel makes such a scenario even more plausible for the eels and is certainly worth investigating further using genome-wide approaches in order to detect genomic regions responsible for the adaptive divergence and hybrid incompatibility found among these eel species.

## Supporting Information

**Table S1A.** Gene name, annotation information, expressionratio, and *q*-values for 146 genes showing significantinteraction between size and species (see Material and Methods fordetails) in two leptocephali samples from two eel species (*A.rostrata* and *A. anguilla*).

**Table S1B.** Details on gene name, annotation information,expression ratio, and *q*-values for 60 genes showingsignificant expression differences between size groups inleptocephali samples from two eel species (see Material and Methodsfor details).
